# Surface settlement law of double-hole pipe-jacking tunnel undercrossing expressway

**DOI:** 10.1038/s41598-023-46364-w

**Published:** 2023-11-07

**Authors:** Meilong Deng, Fei Ding, Yang Liu, Xin Huang, Yu He, Jinpeng Zhao

**Affiliations:** 1 China Construction First Group Corporation Limited, Beijing, 100161 China; 2 Broadvision Engineering Consultants, Yunan, 650041 China; 3 School of Civil Engineering, Beijing Jiaotong University, Beijing, 10044 China

**Keywords:** Civil engineering, Engineering

## Abstract

With the rapid urbanization process in China, there has been a significant development of urban underground spaces. The pipe-jacking method has gained popularity in various projects due to its advantages such as short construction period, adaptability to different soil conditions, and minimal excavation disturbance. This study focuses on a tunnel project involving double-hole rectangular pipe-jacking under an expressway. Utilizing FLAC software, a three-dimensional numerical model was created to analyze the impact of double-hole pipe-jacking construction on surface settlement and deformation of the expressway. The simulation results were validated by comparing them with field monitoring data. The study also investigated the impact of different construction parameters, such as excavation sequence and the angle of underpassing the expressway, on surface settlement. Additionally, the surface settlement curve at the slope bottom of the expressway exhibits asymmetric distribution and an offset center in the settlement trough. It is recommended to carry out the construction of double-hole pipe-jacking in the sequence of "big first and then small". Furthermore, when the included angle between the expressway and the jacking axis is 90°, the impact on surface settlement is minimized. This research provides valuable insights for the construction of double-hole pipe-jacking underpass structures in tunnel engineering.

## Introduction

With the rapid development of urban infrastructure, the construction of underground tunnels has become increasingly important^1,2^. Among the various tunneling methods, the pipe-jacking method has gained significant attention due to its advantages in terms of construction speed, adaptability to different soil conditions, and minimal disturbance to the surrounding environment^3–5^. The pipe-jacking method involves the installation of precast concrete pipes, which are jacked into the ground using hydraulic jacks. This method is particularly suitable for constructing tunnels under existing structures, such as roads, railways, and buildings, as it minimizes the disruption to the surface and reduces the risk of damage to the existing structures^6,7^. One of the critical concerns in the construction of pipe-jacking tunnels is the impact on the surface settlement and deformation of existing structures, which can affect their safety and stability. Therefore, it is essential to study the surface settlement law and its influencing factors to optimize the construction process and ensure the safety and stability of existing structures during the construction of underground tunnels.

The soil disturbance caused by pipe-jacking excavation has been studied theoretically, field tests and laboratory tests. Cheng et al.^5^ proposed a simple approach for characterising tunnel bore conditions based upon pipe-jacking data. Huang et al.^8^ studied the double jacking pipes in Shanghai and proposed a method to guide the jacking construction to protect another tunnel. Jiang et al.^9^ presents a case study of the largest concrete pipe-jacking tunnel project in the world, the sewerage tunnel along Jinshan Lake, Zhenjiang, China. It discussed key technologies such as pipe jacking machine selection, jacking force estimation and control, intermediate jacking station design, grouting process control, tunnel boring machine launch and reception, pipe jacking trajectory control, ventilation and gas monitoring during construction. Liu et al.^10^ believed that during the whole process of pipe-jacking, the settlement displacement of the tunnel mainly experienced three different stages, namely, the initial settlement stage, the rapid lifting stage and the stable lifting stage. Zhang et al.^11,12^ introduced the arc pipe-jacking technology of Gongbei Tunnel. Sun et al.^13^ found that due to the strong soil bearing effect of rectangular pipe-jacking technology, the surface settlement around the pipe-jacking is small. Yuan et al.^14^ believed that the existing pipeline, thickness of overburden layer, grouting slurry and other factors have a significant impact on the ground response during pipe-jacking. Ren et al.^15^ proposed the prediction of surface deformation of pipe-jacking tunnel considering various factors. The above research mainly focuses on the factors affecting the surface settlement of the pipe-jacking tunnel, but the research on the surface settlement of the double-hole rectangular pipe-jacking tunnel and the construction parameters is less.

The double-hole pipe-jacking tunnel has a significant impact on surface settlement, and the construction parameters and layout of the two tunnels also affect the surface. Therefore, this paper aims to analyze the impact of double-hole pipe-jacking construction on surface settlement and deformation of the expressway, focusing on a case study of a double-hole rectangular pipe-jacking under an expressway section of a tunnel project. The simulation results are validated by comparing with field monitoring data. Additionally, the influence of different construction parameters, such as excavation sequence, intersection angle with the expressway, and pipe-jacking distance between the two holes, on surface settlement is investigated.

## Engineering background

### Project overview

**Figure 1:**
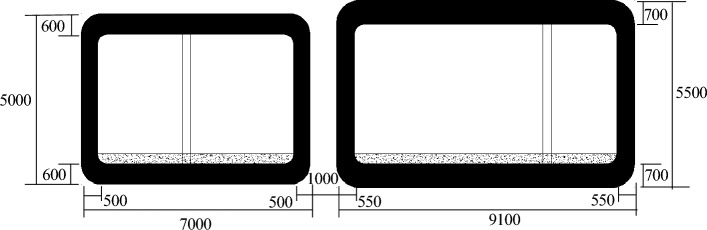
Pipe-jacking section of a tunnel project (unit: mm).

**Figure 2:**
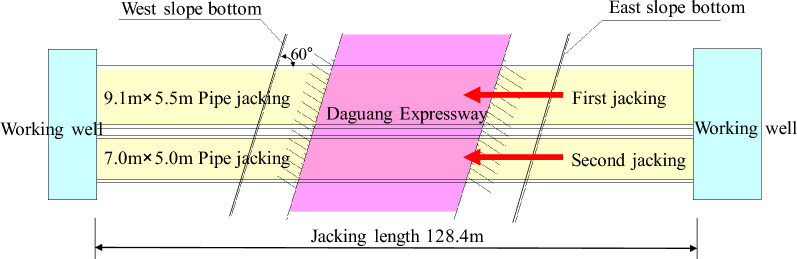
Pipe-jacking plan of a tunnel project.

**Figure 3:**
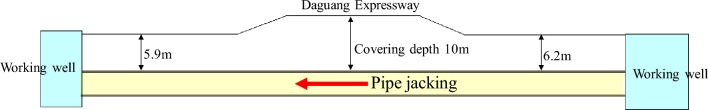
A double-pole tunnel underpasses the expressway.

The rectangular concrete pipe-jacking method is employed for the construction of a tunnel project undercrossing an expressway. Based on the design section size of the tunnel (see Fig. 1), a double-hole pipe-jacking arrangement is adopted. The external dimensions of the two pipe-jacking equipment are 9.1 m × 5.5 m and 7 m × 5 m, respectively. The clear distance between the two holes is 1 m. The jacking length is 128.4 m, with the jacking direction being from east to west up the slope (see Fig. 2). The slope has a gradient of 0.2%. The actual jacking length remains at 128.4 m. The first tunnel to be jacked is the 9.1 m × 5.5 m tunnel, followed by the 7 m × 5 m tunnel. Prefabricated rectangular tunnel linings with reinforced concrete are utilized for the pipe-jacking structure. The concrete strength of the tunnel lining is C50, and it possesses an impermeability grade of P8. The lining interface is of "F" type socket. Figure 3 illustrates the configuration of the tunnel beneath the expressway.

### Pipe-jacking machine

**Figure 4:**
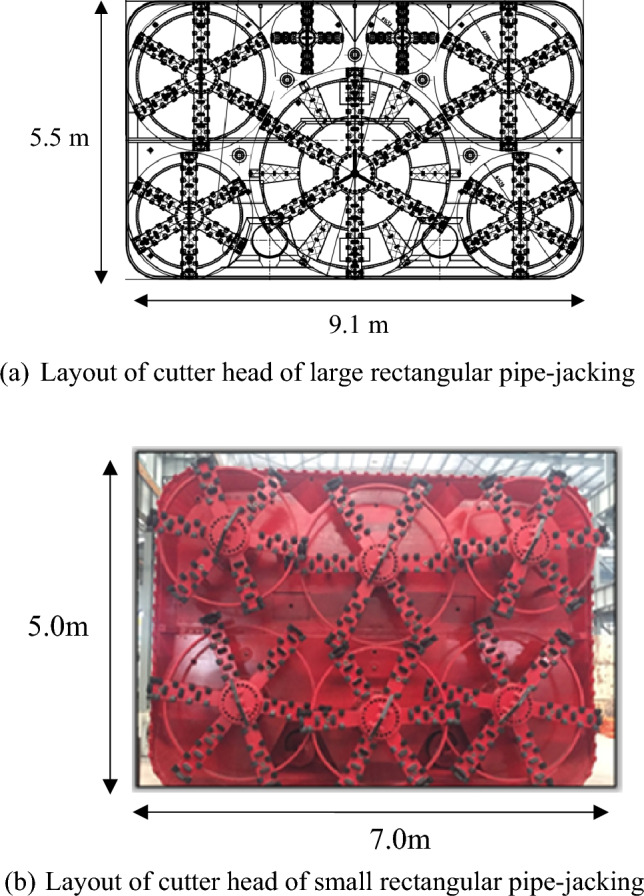
The cutter head of the rectangular pipe-jacking.

Figure 4a illustrates the configuration of the 9.1 m × 5.5 m jacking machine, which features seven cutter heads. The largest cutter head has a diameter of 4200 mm and is powered by eight sets of 30 kW motors, operating at a speed of 1470 r/m. Two medium-sized cutter heads, with diameters of 2980 mm, are equipped with four sets of 30 kW motors, also operating at 1470r/m. Additionally, two medium cutter heads with a diameter of 2520 mm are fitted with three sets of 30 kW motors, and the two smaller cutter heads, with a diameter of 1450 mm, are powered by one 37 kW motor, operating at the same speed of 1470r/m. The total section area of the jacking machine is 49.968m^2^, with a total cutting area of 41.082 m^2^. The cutting rate of the entire cutter head is 82.2%, and the total mixing area is 37.208 m^2^, with a mixing rate of 74.4%. Similarly, Fig. 4b shows the head of the 7.0 m × 5.0 m jacking machine, which is equipped with six cutter heads. The largest cutter head has a diameter of 2980 mm and is powered by three sets of 30 kW motors, operating at a speed of 1470r/m. The two medium-sized cutter heads with diameters of 28000 mm, are fitted with three sets of 30 kW motors, also operating at 1470r/m. Additionally, three small cutter heads with a diameter of 2400 mm, are powered by three sets of 30 kW motors, operating at the same speed of 1470r/m. The total section area of the jacking machine is 34.785m^2^, with a total cutting area of 30.687 m^2^. The cutting rate of the entire cutter head is 88.22%, and the total mixing area is 23.11 m^2^, with a mixing rate of 66.43%.

### Engineering geology

Based on the geological survey data presented in Table 1, the strata encountered during the tunnel crossing consist of clayey silt, silty fine sand, and silty clay. The side wall of the pipe-jacking tunnel primarily traverses the clayey silt layer, where the friction resistance ranges from 48 to 55 kPa. It is crucial to determine the slurry ratio of the thixotropic slurry appropriately during construction to minimize the total jacking force exerted throughout the jacking process. Furthermore, it is worth noting that the elevation of the pipe-jacking is situated above the phreatic layer.

**Table 1:** Strata and its state.

Layer	State
Clay silt layer ②	Yellowish brown to brownish yellow, moderately dense to dense, partially slightly dense, wet, containing mica, iron oxide, mixed with silty clay, sandy silt and thin layer of silty fine sand
Silty fine sand layer ②_1_	mainly composed of mica, quartz and feldspar, mixed with thin layer of silty clay, and partially liquefied
Silty clay layer②_2_	Brownish yellow, plastic, very wet, uneven soil, containing iron oxide, partially mixed with thin layer of clayey silt
Clay silt layer ③	containing mica, manganese oxide and organic matter, partially mixed with silty clay, sandy silt and thin layer of clay

## On-site monitoring of surface displacement of double-hole pipe-jacking tunnel

### Monitoring method and scheme

**Figure 5:**
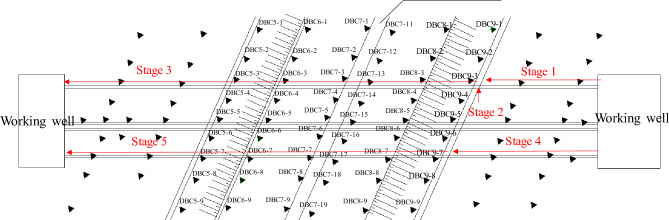
Location of surface settlement points above pipe-jacking tunnels and expressway.

**Figure 6:**
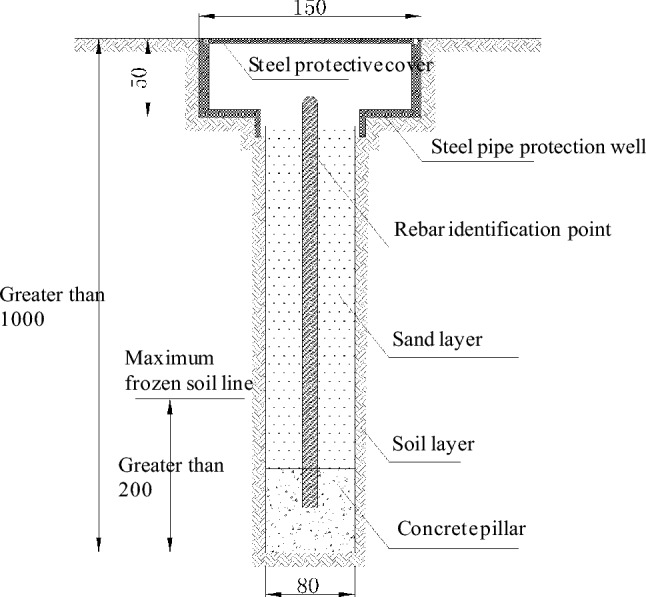
Burial method of surface settlement monitoring points (unit: mm).

The surface settlement displacement is monitored using the Trimble DINI03 electronic level, which has a monitoring accuracy of 0.1 mm. The layout of surface settlement measuring points can be found in Fig. 5. Expressway and surface settlement points are positioned at 10 m intervals along the tunnel direction. Along the expressway's direction outside the tunnel, four rows of measuring points are placed at intervals of 5 m, 5 m, 5 m, and 8 m. The surface settlement measuring point is in the form of a cellar well measuring point, which is created through manual excavation or drilling, as depicted in Fig. 6. According to the relevant specifications for tunnels in China^16^, the surface settlement of expressways must be kept within −15 mm to + 5 mm, while other locations are controlled at −30 mm to + 10 mm.

### Monitoring result

#### Surface settlement along the expressway

The construction process is divided into five stages, as depicted in Fig. 6. In the first stage, the first pipe-jacking tunnel is jacked towards the monitoring section of the east slope of the expressway. The second stage involves jacking the first pipe-jacking tunnel to the monitoring section located at the east slope of the expressway. In the third stage, the first pipe-jacking tunnel is connected. The fourth stage occurs before the second pipe-jacking tunnel is jacked to the monitoring section. Finally, in the fifth stage, the two pipe-jacking tunnels are connected.

**Figure 7:**
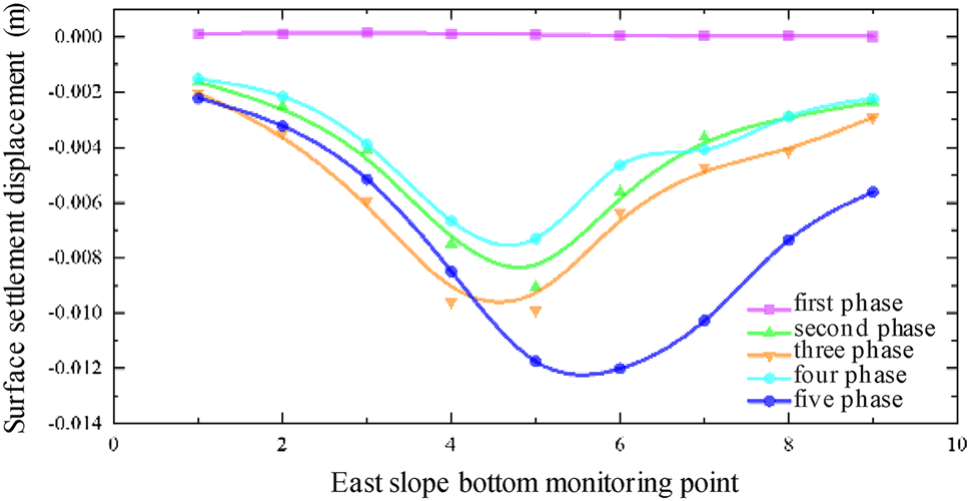
Monitoring point data along the east slope bottom of the expressway.

The settlement data along the east slope bottom of the expressway is presented in Fig. 7. It is evident from the figure that the settlement displacement of the DBC9-5 measuring point above the pipe-jacking axis is the highest, measuring 11.74 mm, after the first pipe-jacking is connected. The settlement decreases continuously along the direction of both ends of the expressway, forming a symmetrical "settlement trough" curve. During the excavation of the second pipe-jacking, several factors such as the grouting pressure of the second pipe-jacking, the superposition effect of the second pipe-jacking, and the adjustment of the pressure of the soil bin influence the settlement. As a result, the center of the surface settlement trough deviates towards the direction of the subsequent small pipe-jacking, resulting in an asymmetric distribution of the settlement curve.

**Figure 8:**
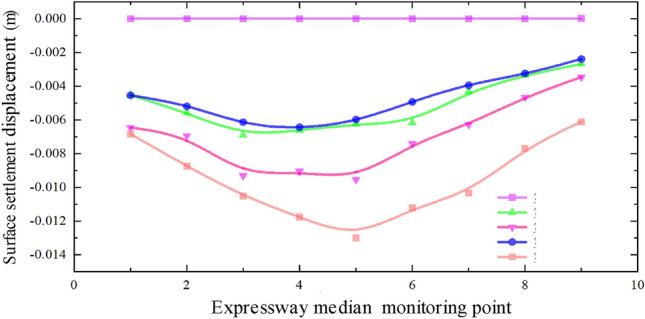
Data of monitoring points along the median of expressway.

**Figure 9:**
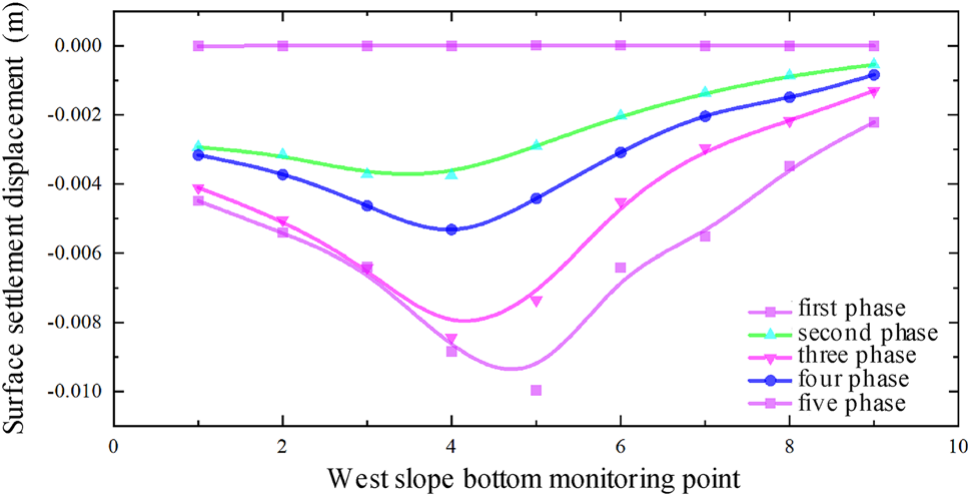
Monitoring point data along the west slope bottom of the expressway.

Figure 8 illustrates the data from monitoring points along the median of the expressway, while Fig. 9 displays the monitoring point data along the west slope bottom of the expressway. It can be observed that the settlement displacement relationship curves in Figs. 7, 8 and 9 exhibit some variations. The surface settlement displacement relationship curve in the median of the expressway demonstrates better symmetry compared to the monitoring points at the slope bottom of the expressway. It can be attributed to the reinforced subgrade of the expressway, which experiences less influence from the superposition effect of the double-hole pipe-jacking construction.

In the surface settlement displacement relationship curve at the east slope bottom of the expressway, the settlement values of the measuring points on the large pipe-jacking side are slightly lower than those on the small pipe-jacking side. Conversely, in the surface settlement displacement relationship curve at the west slope bottom of the expressway, the settlement values of the measuring points on the large pipe-jacking side are slightly higher than those on the small pipe-jacking side. Additionally, the center of the settlement trough at each measuring point at the bottom of the west slope consistently inclines towards the first large jacking direction.

Based on the actual project site conditions, the reasons for the observed asymmetry in the settlement displacement relationship curve are analyzed. Firstly, the position of the No. 5 monitoring point in each column of all monitoring arrays is directly above the outer wall of the large-section pipe-jacking between the two pipe-jacking tunnels, while the No. 4 monitoring point is located in the direction of the large-section pipe-jacking axis. However, the overall layout of the measurement points is relatively symmetrical about the common section of the two pipe-jacking tunnels, rather than a specific pipe-jacking axis direction. Secondly, due to the different dimensions of the two pipe-jacking tunnels, the range and degree of soil disturbance caused by pipe-jacking with different size sections varies, and there are also factors of disturbance superposition, resulting in the asymmetry of the measured data. Furthermore, there is a certain angle between the layout of the expressway and the axis of the pipe-jacking tunnels. The dead weight of the subgrade on the expressway and the vehicle load on the pavement also have a certain degree of influence on the settlement displacement of the surface.

#### Surface settlement along the expressway

**Figure 10:**
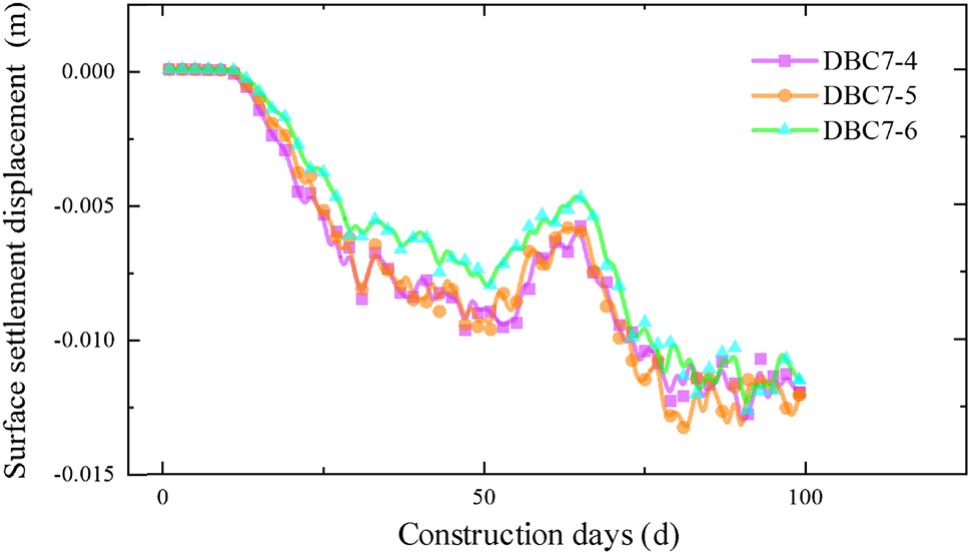
Monitoring point data of expressway isolation zone.

**Figure 11:**
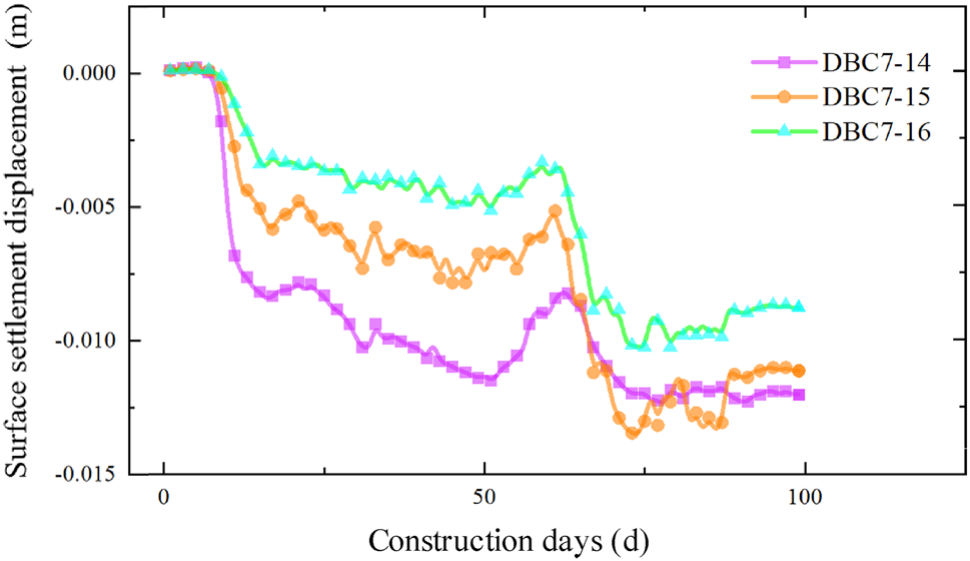
Monitoring point data of expressway isolation zone.

**Figure 12:**
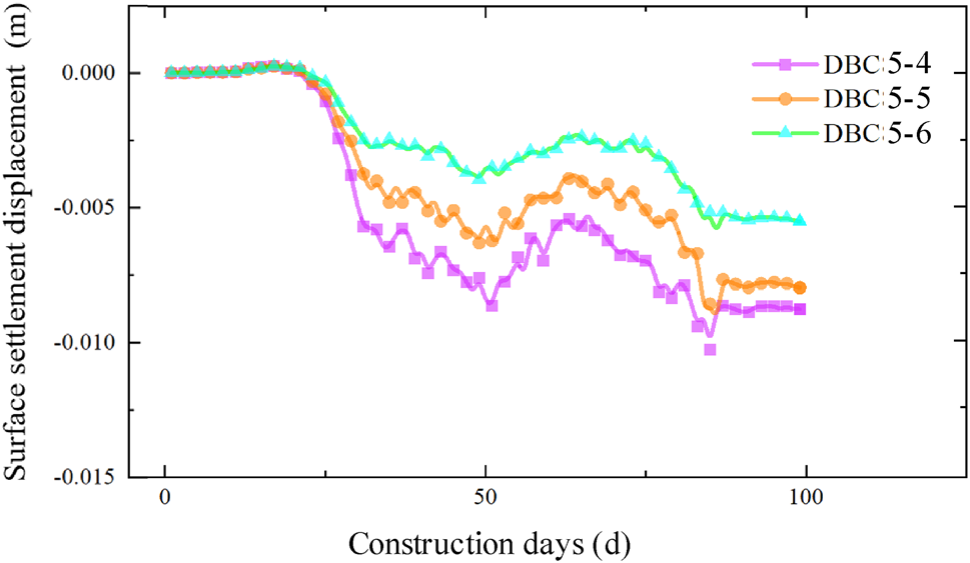
Monitoring point data of slope bottom on the west side of expressway.

**Figure 13:**
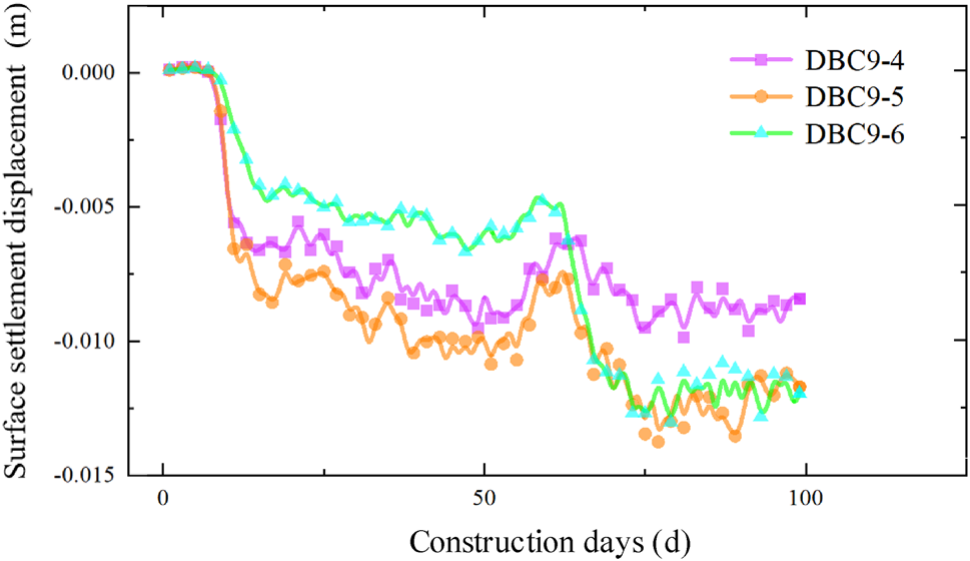
Monitoring point data of slope bottom on the east side of expressway.

The settlement data for each section as the pipe-jacking machine passes through, obtained from the monitoring points distributed along the pipe-jacking axis, are presented in Figs. 10, 11, 12 and 13. Prior to the arrival of the pipe-jacking machine at the section where the monitoring point is located, the ground surface experiences a slight uplift in settlement displacement. It can be attributed to the soil mass uplifting under the squeezing of the top thrust from the pipe-jacking machine. As the pipe-jacking machine reaches the section, the ground surface undergoes a rapid settlement displacement. Subsequently, after passing the section, the ground surface continues to settle but tends to stabilize until the large pipe-jacking operation is initiated. When the subsequent small pipe-jacking operation is conducted, the overall trend remains relatively unchanged, but there is a slight reduction in the overall settlement value, representing the slight uplift observed in the initial stage. The "uplift value" at this stage is slightly greater than the uplift value when the large pipe-jacking operation is conducted first. As the pipe-jacking machine passes through the monitoring point, the monitoring value experiences a rapid increase, and the final settlement displacement of the ground surface exhibits an overall settlement, with a maximum settlement value of 13.78 mm.

## Study on the influence of different construction parameters of double-hole pipe-jacking tunnel on surface settlement

### Establishment and verification of numerical model

#### Establishment of numerical model

**Figure 14:**
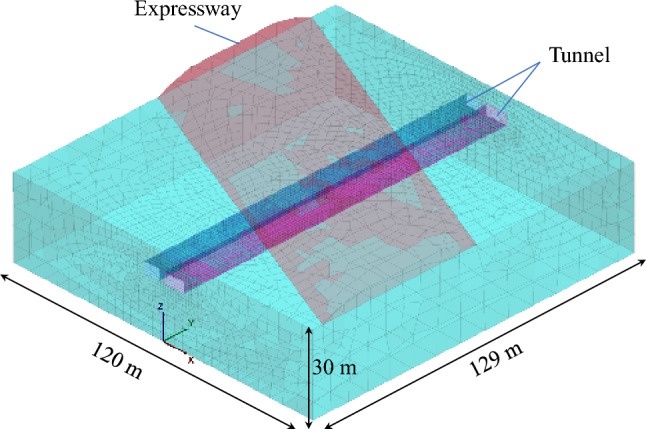
Model meshing.

The 3D model presented in Fig. 14 depicts the established model. The X-axis direction represents the normal direction of the pipe-jacking axis, the Y-axis direction represents the forward direction of the pipe-jacking axis, and the negative direction of the Z-axis represents the force of gravity. To minimize the influence of boundary effects, the model size encompasses the area affected by the pipe-jacking construction disturbance, extending 120 m in the X-axis direction, 129 m in the Y-axis direction, and 30 m in the Z-axis direction. Additionally, the expressway subgrade has a height of 4.3 m.

The Mohr–Coulomb model is employed to simulate the behavior of the rock and soil mass, while an elastic model is utilized to represent the pipe-jacking machine and reinforced concrete lining. Furthermore, an empty model is adopted to simulate the excavation process of the pipe-jacking project. The physical and mechanical parameters of the strata are presented in Table 2.

**Table 2:** Physical and mechanical parameters of different strata.

Name	Elastic modulus E(MPa)	Density (g/cm^3^)	Internal friction angle φ (°)	Cohesion c(kPa)	Poisson's ratio v	Layer thickness (m)
Plain fill layer	17	1.72	22	21	0.35	0.79
Sandy silt layer	23	1.85	31	31.5	0.34	4.7
Clay silt layer 1	25	1.91	25	19.8	0.3	7.4
Clay silt layer 2	27	1.94	26	20.6	0.3	4.0
Silty fine sand	24	1.92	28	19.5	0.32	2.6
Silty fine sand layer	26	19.6	29	41.4	0.26	10.51

Due to the small thickness and proximity to the outer wall of the pipe-jacking tunnel, the grouting layer is also simulated using an elastic model. The elastic modulus for the grouting layer is set at 0.1 GPa, with a Poisson's ratio of 0.2 and a weight of 2300 kg/m^3^. In this project, the lining material used for pipe-jacking is C50 reinforced concrete. Considering the reduction in rigidity caused by the connection of linings, a reduction factor of 0.8 is applied. The elastic modulus for the tunnel lining is 28.0 GPa, with a Poisson's ratio of 0.2 and a weight of 2500 kg/m^3^.

The pipe-jacking construction process involves several stages, including jacking, grouting, drag reduction, and slag discharge, all of which have an impact on the settlement displacement of the ground surface. Due to the complexity of the construction process, it is not feasible to fully replicate it in the numerical model. Therefore, certain simplifications have been made. To simulate the jacking force at the excavation surface, a uniform load of 200 kPa is applied in the Y direction on the excavation surface. The grouting pressure of 150 kPa is simulated by applying an outward uniform load on the four sides of the grouting layer. Additionally, to simulate the frictional resistance, a shear stress of −10 kPa is applied in the Y direction between the segment and the soil. In this project, the length of the prefabricated segments is 1.5 m. Consequently, the distance of each excavation step is set as 1.5 m.

The numerical model calculation steps are as follows: (1) Assign values to the soil layers and calculate the initial geostress state and initial displacement state without excavation. In the subsequent process simulation, the initial displacement value is set to zero after balancing. (2) Model the soil mass, pipe-jacking head, and lining as empty models for the first excavation step, indicating its completion. Establish the lining structural elements to simulate the pipe joints and calculate the balance. (3) Apply jacking force on the excavation face, excavate the next 1.5 m step, and apply grouting pressure on the mud layer behind the excavation step. (4) Repeat steps 2 and 3 until the pipe-jacking construction is fully excavated.

#### Model validation

**Figure 15:**
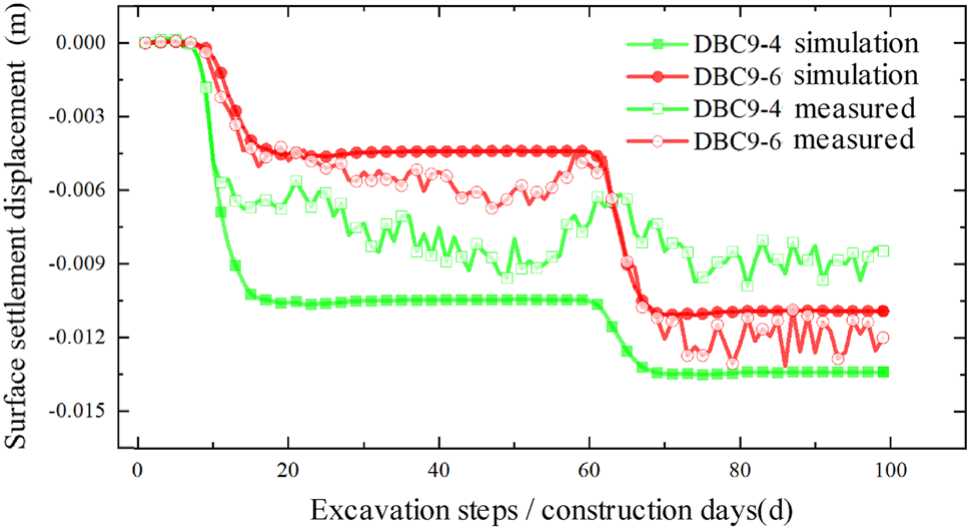
Comparison between simulated value and measured value of slope bottom on the east side of expressway.

**Figure 16:**
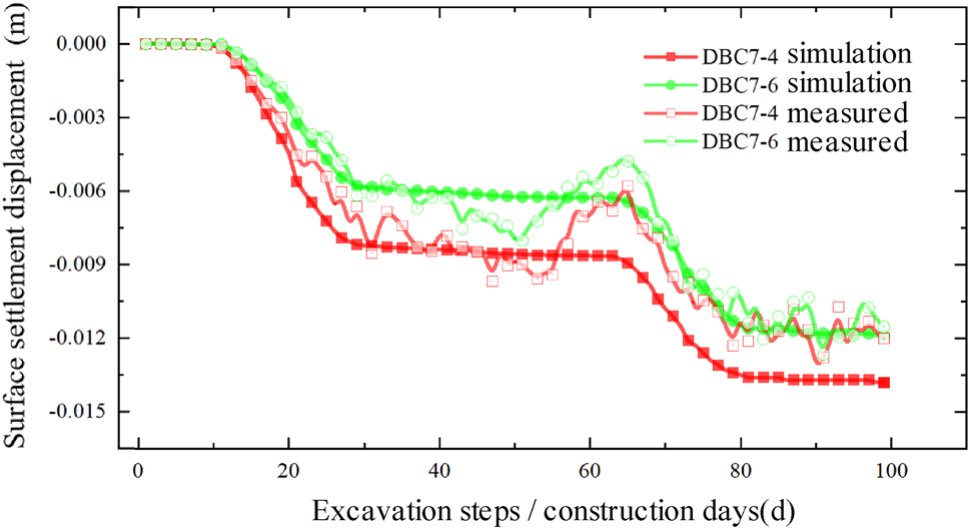
Comparison between simulated value and measured value of expressway median.

**Figure 17:**
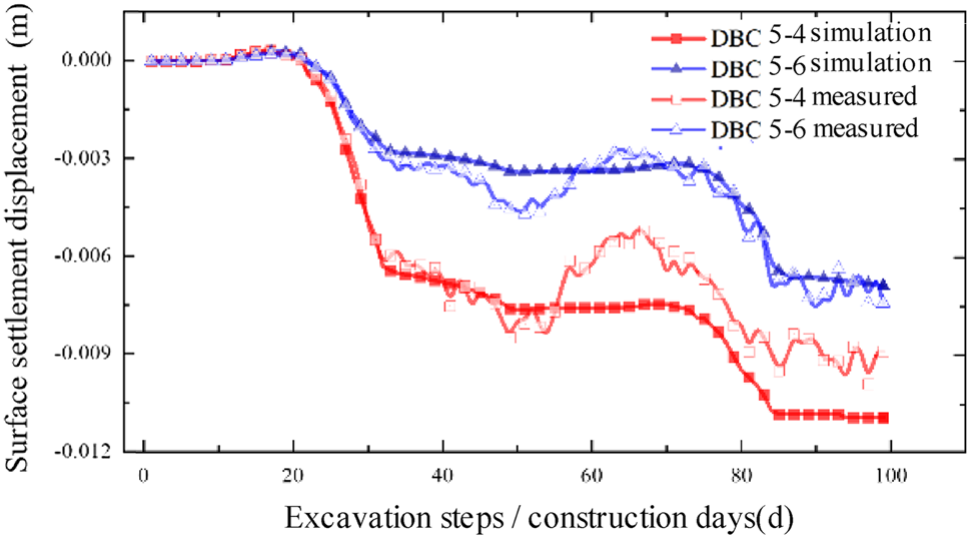
Comparison between simulated value and measured value of slope bottom on the west side of expressway.

By comparing and analyzing the settlement values obtained from the numerical model with the actual monitoring data, we can assess the accuracy of the model and investigate the effects of pipe-jacking construction on soil disturbance. This analysis will provide a valuable reference for formulating effective measures to control surface settlement. Figures 15, 16, and 17 illustrate the numerical comparison of slope bottom settlements on the east side, median, and west side of the expressway.

Upon comparison, it was observed that the simulated values and measured values generally exhibited good agreement, indicating that the model's accuracy is reasonably reliable. This finding can provide valuable insights for similar projects in the future. However, it is worth noting that there is a certain degree of error between the numerical simulation values and the measured values. In the model simulation, the vehicle dynamic load on the expressway was approximated by applying a normal stress in the negative direction of the Z-axis on the expressway, utilizing a uniformly distributed static load. While the daily average traffic flow on the expressway is substantial, there are periods of low traffic flow where the actual load is relatively small, resulting in significant load fluctuations. Moreover, the measured data may fluctuate within a certain range due to factors such as weather conditions, instrument errors, and operational mistakes at the construction site. Regarding the relative "heave" discrepancy, during the actual construction process, the foundation pit construction of the pipe-jacking starting shaft requires the installation of retaining piles to reinforce the surrounding soil. This process disturbs the initial soil conditions and alters the soil's original properties, leading to a relatively upward "heave" during the jacking process.

### Law of surface settlement in different construction sequences of double-hole pipe

**Figure 18:**
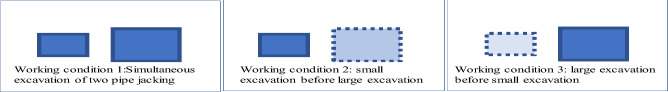
Working conditions of different construction sequences.

The various section sizes of pipe-jacking have three distinct construction sequence conditions. This section investigates the influence of these different construction sequence conditions on surface settlement and displacement, as illustrated in Fig. 18.

#### Working condition 1: simultaneous jacking

**Figure 19:**
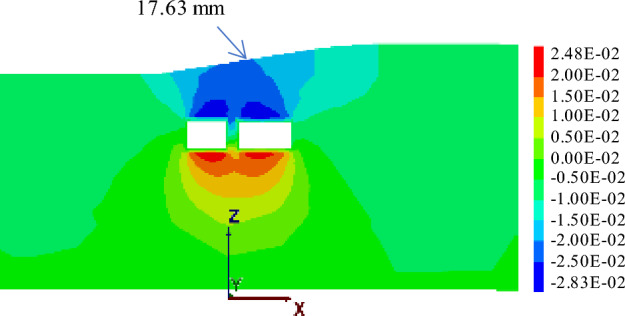
Displacement of two tunnels excavated simultaneously to the monitoring section.

When the pipe-jacking machine is advanced into the central separation zone of the expressway, the maximum ground surface settlement displacement is 14.37 mm. Continuing with the jacking process, upon completion, as depicted in Fig. 19, the maximum surface settlement displacement at the expressway pavement reaches 17.63 mm.

#### Working condition 2: small first and then big

**Figure 20:**
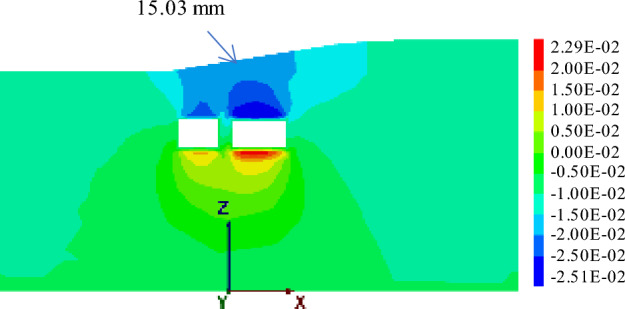
Displacement when jacking " small first and then big " to the monitoring section.

In previous construction experiences, it has been observed that larger tunnel diameters of underground caverns result in greater disturbance to the surrounding soil, leading to a greater impact on surface settlement displacement. As shown in Fig. 20, under such working conditions, the maximum surface settlement displacement reaches 15.03 mm. In contrast, when small section pipe-jacking is employed to reach the expressway isolation zone, the maximum surface settlement displacement is reduced to 5.46 mm during the jacking process and 6.21 mm upon completion. However, when using large-section pipe-jacking to reach the same isolation zone, the maximum surface settlement displacement is 13.39 mm.

#### Working condition 3: big first and then small

**Figure 21:**
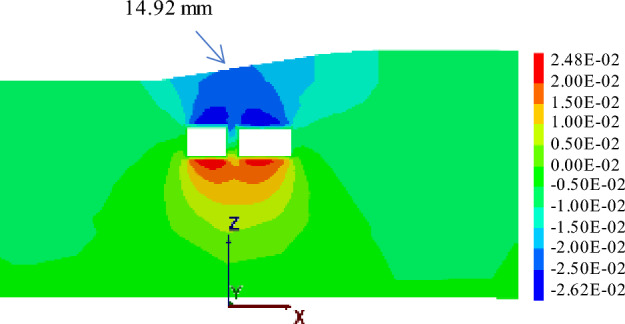
Displacement when jacking "big first and then small" to the monitoring section.

Figures 21 shows the displacement when jacking "big first and then small" to the monitoring section. Under these conditions, the maximum surface settlement displacement measures 14.92 mm. Upon reaching the expressway isolation zone, the large-section pipe-jacking results in a maximum surface settlement displacement of 0.98 mm. Once the large-section pipe-jacking is completed, the maximum surface settlement displacement reaches 8.81 mm. Subsequently, when the small-section pipe-jacking is carried out to reach the expressway isolation zone, the maximum surface settlement displacement amounts to 8.89 mm.

Based on the analysis of three different working conditions, it has been determined that the simultaneous jacking of two pipe-jacking tunnels is the least favorable, resulting in the largest vertical settlement displacement of the ground surface. Conversely, the most optimal approach is to follow the jacking sequence of "big first and then small". Under this sequence, the maximum settlement displacement of the ground surface reaches 14.92 mm, which is only 0.11 mm higher than the maximum settlement observed in the sequence of "small first and then big". These findings can offer valuable insights for future projects of a similar nature.

### Influence of different spacing between two pipe jacks on surface settlement displacement

The subsequent pipe-jacking construction will have a cumulative impact on the disturbance of the surrounding soil. One notable feature is the pronounced cumulative effect on settlement changes, while the cumulative effect on the influence range is not particularly evident. In this specific engineering project, the outer diameter interval between the two pipe-jackings is 1 m, due to site constraints. Building upon this, we explore the influence of different outer wall intervals between two pipe-jackings with varying sections on the settlement displacement of the ground surface, disregarding the limitations imposed by the construction site.

#### The interval between the outer walls of two jacking pipes is 0.5 m

**Figure 22:**
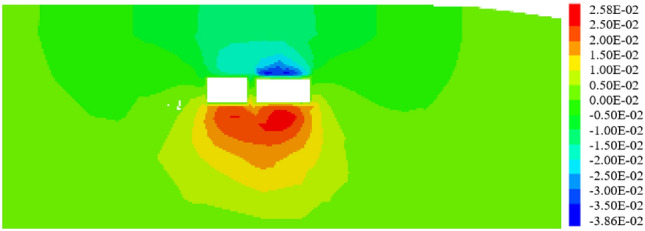
Section at 64 m when two tunnels are connected at an interval of 0.5 m.

When the interval between pipe-jackings with different cross sections is reduced to 0.5 m, the jacking sequence of large section pipe followed by small section pipe is employed, as illustrated in Fig. 22. Under these conditions, the maximum surface settlement displacement measures 14.30 mm. Upon reaching the expressway isolation zone, the large-section pipe-jacking results in a surface settlement displacement of 6.96 mm. Once the large-section pipe-jacking is completed, the surface settlement displacement reaches 8.76 mm. Subsequently, when the small-section pipe-jacking is carried out to reach the same isolation zone, the surface settlement displacement amounts to 12.36 mm.

#### The interval between the outer walls of two jacking pipes is 8 m

**Figure 23:**
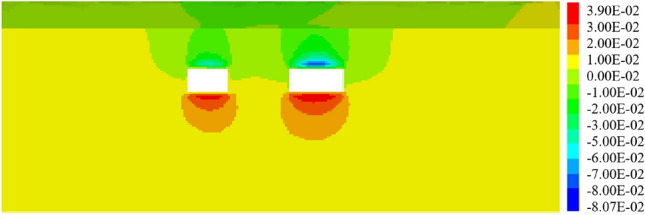
Section at 64 m when two tunnels are connected at an interval of 8 m.

In order to analyze the impact on the settlement displacement of the ground surface, the outer wall spacing of the two pipe-jackings is considered to be equal to the average width of the two sections, which are 9.1 m and 7.1 m, respectively. The construction sequence of "big first and then small" is followed, as depicted in Fig. 23. Under this specific working condition, the maximum settlement displacement of the surface is measured to be 13.58 mm. Upon reaching the monitoring point of the expressway isolation zone, the settlement displacement of the surface during the jacking of the large-section pipe amounts to 7.76 mm. Once the large-section pipe-jacking is completed, the settlement displacement of the surface is recorded as 8.82 mm. Subsequently, when the small-section pipe-jacking reaches the monitoring point of the expressway isolation zone, the surface settlement displacement is measured at 12.39 mm. These findings provide valuable insights for similar projects in the future.

**Figure 24:**
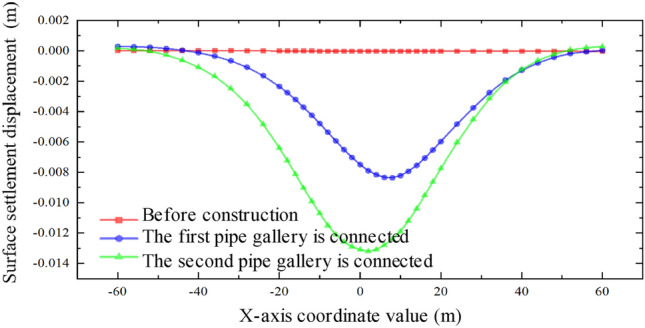
Relation curve of settlement displacement of ground surface at different time in the intermediate isolation zone with an interval of 8 m.

By analyzing the results, it is observed that when the interval between the two pipe-jackings is 8 m, the cumulative disturbance effect on the soil mass is weakened, while the cumulative effect on the disturbance range of the soil mass is enhanced, leading to an enlarged influence range on the expressway. As depicted in Fig. 24, the maximum surface settlement occurs above the axis of the large-section pipe-jacking after the completion of the first pipe-jacking. Upon connecting the second pipe-jacking, the maximum surface settlement displacement shifts towards the direction of the small-section pipe-jacking. However, the central point of the final settlement trough remains close to the side of the large-section pipe-jacking. It is also noted that the larger the section of the pipe-jacking, the greater the impact on the surface settlement displacement.

#### The interval between the outer walls of two jacking pipes is 16 m

**Figure 25:**
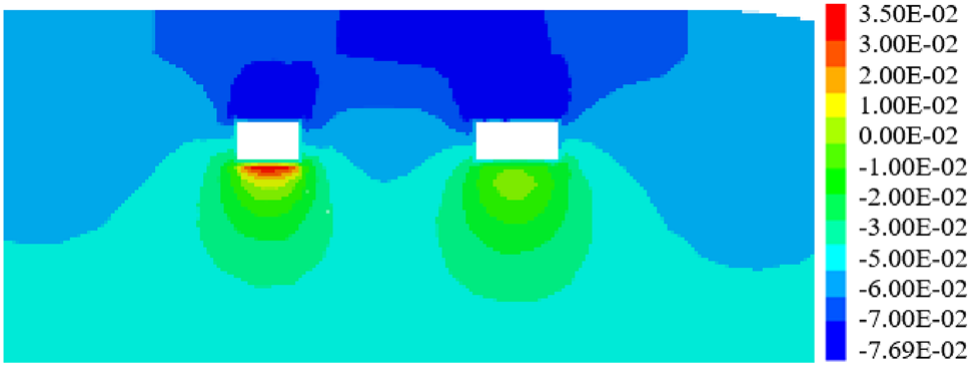
Section at 64 m when two tunnels are connected at an interval of 16 m.

The widths of the two jacking tubes are 9.1 m and 7.1 m respectively. The spacing between the outer walls of the two pipe-jackings is equal to the sum of their widths. The jacking sequence follows the "big first and then small" construction approach, as depicted in Fig. 25. Under these working conditions, the maximum settlement displacement of the ground surface is measured at 11.57 mm, with a coordinate of 8 m from the origin. When the large-section pipe-jacking reaches the monitoring point of the expressway isolation zone, the settlement displacement of the ground surface amounts to 7.87 mm. After the completion of the large-section pipe-jacking, the settlement displacement of the ground surface is recorded as 9.00 mm. Upon reaching the monitoring point of the expressway isolation zone, the small-section pipe-jacking results in a surface settlement of 10.85 mm.

**Figure 26:**
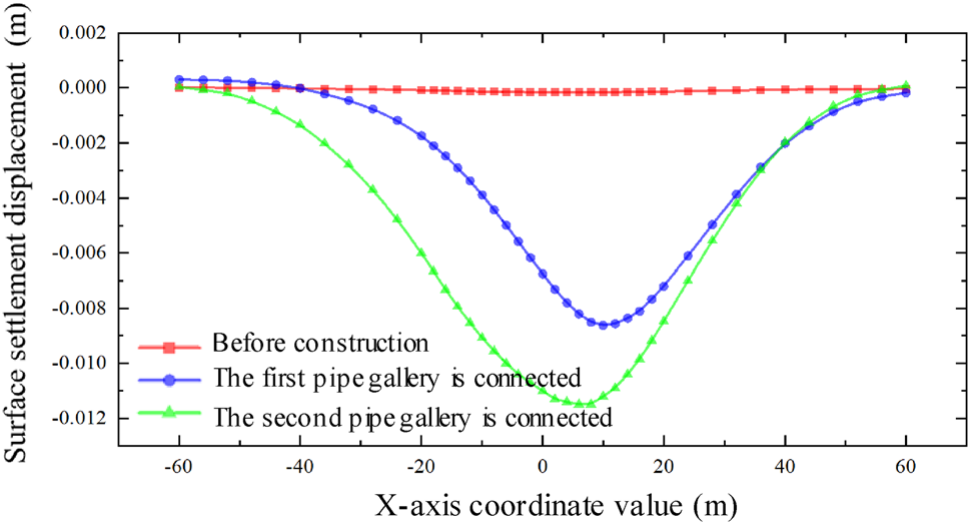
Surface settlement relation curve with interval of 16 m.

From Fig. 26, it is evident that when the spacing between the outer walls of the two jacking pipes is 16 m, the cumulative effect on the disturbance range is further amplified, potentially impacting the entire expressway pavement in the model. However, the cumulative effect on the disturbance degree is weakened. The center point of the final settlement trough continues to shift towards the side of the large pipe-jacking. Interestingly, the maximum vertical surface settlement is reduced in this scenario. These findings provide valuable insights for understanding the behavior of the soil mass during the jacking process and can be useful for optimizing future pipe-jacking projects.

**Figure 27:**
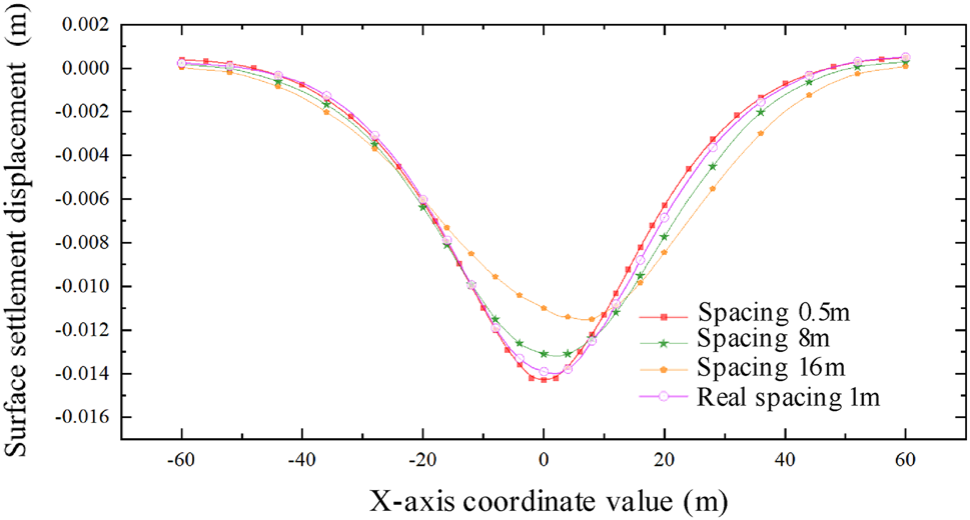
Surface settlement relation curve at different intervals.

From Fig. 27, it is evident that as the spacing between the two different sections increases, the maximum settlement displacement of the ground surface gradually decreases. This indicates that the superposition effect of the surface settlement caused by the double-hole pipe-jacking is weakened. Furthermore, the center of the settlement trough consistently shifts towards the side of the large pipe-jacking. Additionally, the disturbance range of the soil mass continuously expands. However, it is worth noting that the sensitivity of the impact on the disturbance range is less significant compared to the sensitivity of the impact on the disturbance degree.

### Influence of different angle between expressway and pipe-jacking on settlement displacement of ground surface

In the actual project described in this paper, the included angle between the expressway direction and the axis direction of the pipe-jacking is approximately 60°. The plane position relationship determines that the distance from the jacking of the large-section pipe to the expressway is shorter than the distance from the jacking of the small-section pipe to the expressway. The distance between the outer walls of the double-hole pipe-jacking is 1 m, based on the jacking sequence of first large and then small. With this information as a foundation, the aim is to explore the influence of different included angles between the expressway and pipe-jacking on the settlement displacement of the ground surface. The findings will provide valuable insights for the design of similar projects in the future.

#### The included angle between expressway and pipe-jacking axis is 90°

**Figure 28:**
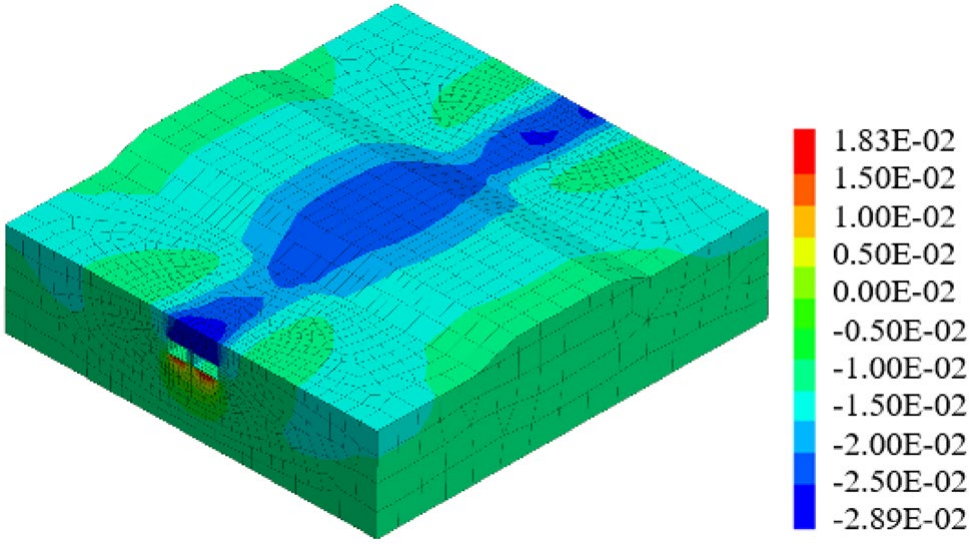
Displacement stereogram at the intersection angle of 90° between tunnel and expressway.

When the expressway is perpendicular to the pipe-jacking axis and the included angle is 90°, the jacking should be carried out following the construction sequence of "big first and then small," as depicted in Fig. 28. Under these working conditions, the maximum settlement displacement of the surface is 14.90 mm. When the large-section pipe-jacking reaches the monitoring point of the expressway isolation zone, the surface settlement is measured at 5.82 mm. After the completion of the large-section pipe-jacking, the surface settlement is recorded at 9.38 mm. Finally, when the small-section pipe-jacking reaches the monitoring point of the expressway isolation zone, the surface settlement is observed at 12.31 mm.

#### The included angle between expressway and pipe-jacking axis is 60°

**Figure 29:**
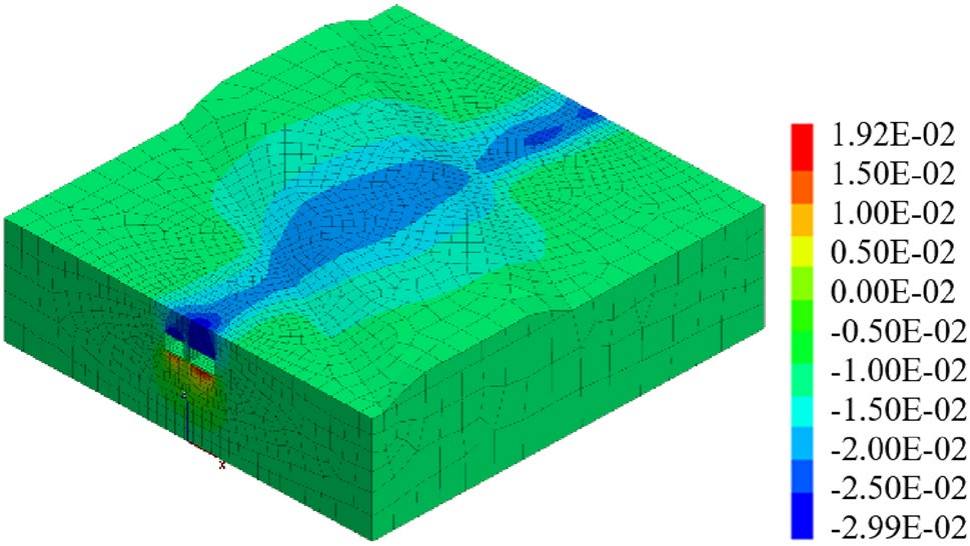
Displacement stereogram at the intersection angle of 60° between tunnel and expressway.

When the included angle between the expressway and the pipe-jacking axis is 60°, the jacking should be carried out following the construction sequence of "big first and then small," as depicted in Fig. 29. Under these working conditions, the maximum settlement displacement of the surface is measured at 14.92 mm. When the large-section pipe-jacking reaches the monitoring point of the expressway isolation zone, the settlement displacement of the surface is recorded at 0.98 mm. After the completion of the large-section pipe-jacking, the settlement displacement of the surface is observed at 8.81 mm. Finally, when the small-section pipe-jacking reaches the monitoring point of the expressway isolation zone, the settlement displacement of the surface is measured at 8.89 mm.

#### The included angle between expressway and pipe-jacking axis is 30°

**Figure 30:**
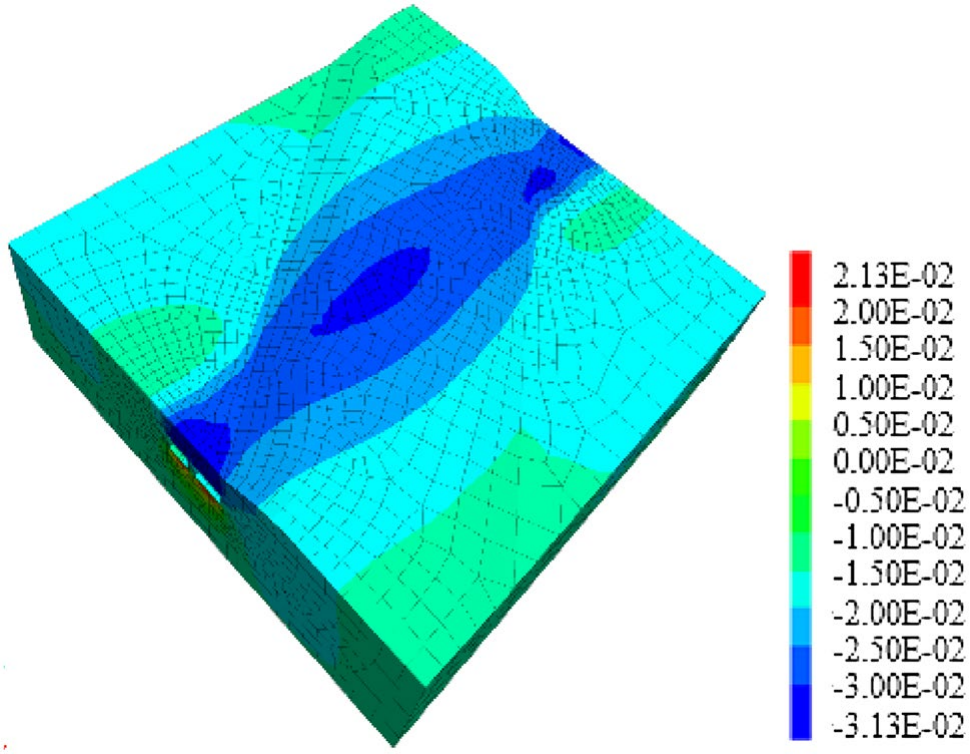
Displacement stereogram at the intersection angle of 60° between tunnel and expressway.

Similarly, when the included angle between the expressway and the pipe-jacking axis is 30°, the jacking should be carried out following the construction sequence of "big first and then small," as shown in Figs. 30, 32. Under these working conditions, the maximum settlement displacement of the surface is recorded at 16.03 mm. When the large-section pipe-jacking reaches the monitoring point of the expressway isolation zone, the settlement displacement of the surface is measured at 8.66 mm. After the completion of the large-section pipe-jacking, the settlement displacement of the surface is observed at 9.94 mm. Finally, when the small-section pipe-jacking reaches the monitoring point of the expressway isolation zone, the settlement displacement of the surface is measured at 14.54 mm.

**Figure 31:**
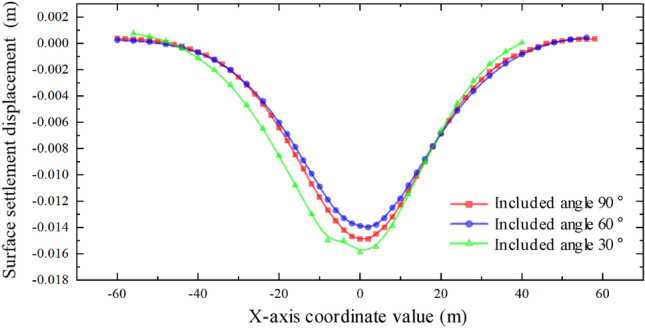
Two through displacement curves with different included angles.

**Figure 32:**
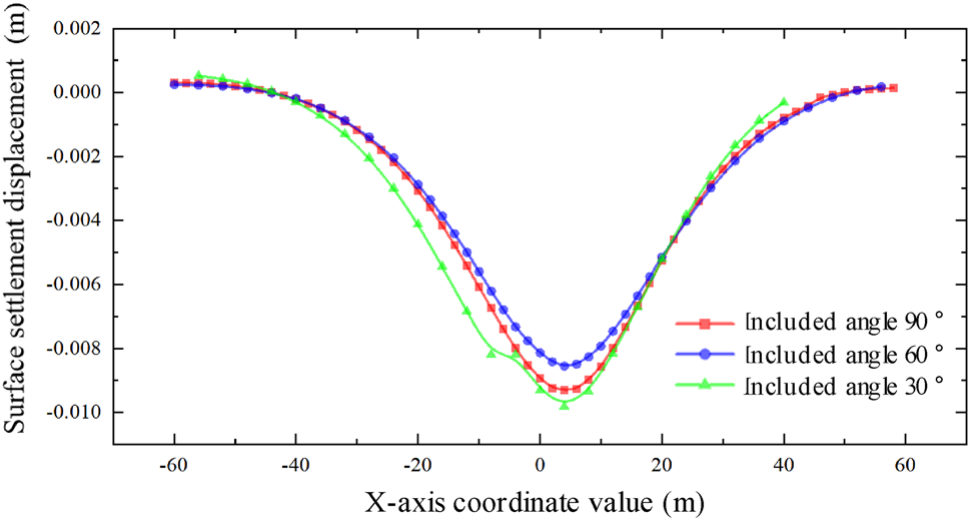
One through displacement curve with different included angles.

Through the analysis of the displacement of the middle isolation belt of the expressway after the completion of two and one pipe-jacking under the conditions that the included angles of the expressway and the jacking axis are 30°, 60°, and 90°, as depicted in Figs. 31 and 32, it is found that the settlement displacement of the surface on the expressway is the largest at 16.03 mm when the included angle is 30°. The surface displacement is 14.92 mm when the included angle is 60°, and the settlement displacement on the expressway is the smallest at 14.90 mm when the included angle is 60°.

## Conclusions

The aim of this study was to investigate the soil settlement disturbance caused by double-hole rectangular pipe-jacking under an expressway. This was accomplished through finite element analysis software and analysis of field measured data. The following conclusions can be drawn:

1. The curve of pavement settlement during double-hole pipe-jacking construction does not completely adhere to the law of complete symmetry of settlement trough curve, but it has a minor impact on the disturbance range of soil. The variation rules of surface settlement can be summarized as follows: slight uplift stage, rapid settlement stage, continuous settlement stage, and stable stage.
2. By comparing the numerical model results with the measured values, the analysis of measured data confirms the relevant conclusions. However, there are some differences: the simulated values are generally larger than the measured values, especially in the stage of stable settlement after construction. The measured values also exhibit slightly larger fluctuations compared to the simulated values. Additionally, after the completion of the first pipe-jacking and penetration, the settlement displacement of the ground surface is initially upward during the second pipe-jacking, with a relative "uplift" greater than the simulated value.
3. It was concluded that simultaneous double-hole pipe-jacking is the most unfavorable working condition. The final settlement value of the "big first and then small" construction sequence is smaller than that of the "small first and then large" sequence, making the "big first and then small" jacking sequence the optimal construction sequence.
4. When the angle between the expressway and the tunnel are 30°, 60°, and 90° respectively, the included angle of 30° has the largest influence range on surface settlement, resulting in the largest surface settlement value. Conversely, when the included angle is 90°, the range of influence on surface settlement is the smallest, with the smallest surface settlement value. At this angle, the "settlement trough" curve is relatively symmetrical.

## Data Availability

The datasets used and analysed during the current study available from the corresponding author on reasonable request.
